# Application of advanced diffusion models from diffusion weighted imaging in a large cohort study of breast lesions

**DOI:** 10.1186/s12880-023-01005-6

**Published:** 2023-04-11

**Authors:** Ying Ji, Junqi Xu, Zilin Wang, Xinyu Guo, Dexing Kong, He Wang, Kangan Li

**Affiliations:** 1 Department of Radiology, Shanghai General Hospital, Shanghai Jiaotong University School of Medicine, No. 650, New Songjiang Road, Shanghai, 201620 China; 2 Institute of Science and Technology for Brain-Inspired Intelligence, Fudan University, No. 220, Handan Road, Shanghai, 200433 China; 3 School of Mathematical Sciences, Zhejiang University, No. 866, Yuhangtang Road, Zhejiang, 310027 China

**Keywords:** Breast lesions, B-value, Parameters, Prognostic factors, Molecular subtypes

## Abstract

**Background:**

To evaluate multiple parameters in multiple b-value diffusion-weighted imaging (DWI) in characterizing breast lesions and predicting prognostic factors and molecular subtypes.

**Methods:**

In total, 504 patients who underwent 3-T magnetic resonance imaging (MRI) with T1-weighted dynamic contrast-enhanced (DCE) sequences, T2-weighted sequences and multiple b-value (7 values, from 0 to 3000 s/mm^2^) DWI were recruited. The average values of 13 parameters in 6 models were calculated and recorded. The pathological diagnosis of breast lesions was based on the latest World Health Organization (WHO) classification.

**Results:**

Twelve parameters exhibited statistical significance in differentiating benign and malignant lesions. alpha demonstrated the highest sensitivity (89.5%), while sigma demonstrated the highest specificity (77.7%). The stretched-exponential model (SEM) demonstrated the highest sensitivity (90.8%), while the biexponential model demonstrated the highest specificity (80.8%). The highest AUC (0.882, 95% CI, 0.852–0.912) was achieved when all 13 parameters were combined. Prognostic factors were correlated with different parameters, but the correlation was relatively weak. Among the 6 parameters with significant differences among molecular subtypes of breast cancer, the Luminal A group and Luminal B (HER2 negative) group had relatively low values, and the HER2-enriched group and TNBC group had relatively high values.

**Conclusions:**

All 13 parameters, independent or combined, provide valuable information in distinguishing malignant from benign breast lesions. These new parameters have limited meaning for predicting prognostic factors and molecular subtypes of malignant breast tumors.

## Background

Breast cancer is the most common malignancy among women [1]. The latest research has shown that female breast cancer has transcended lung cancer as the most commonly diagnosed cancer [2]. With the development and popularization of contemporary imaging technology, breast lesions, as well as their pathological subtypes, which are critically important for treatment plan-making, can be more precisely predicted. Mammography, ultrasound and magnetic resonance imaging (MRI) are the most commonly used examination methods in the clinic, and MRI is considered as an essential method for deducing the nature of the mass.

Breast dynamic contrast-enhanced (DCE) MRI has been commonly used to clarify tumor shape and blood supply, and it is sometimes applied in preoperative evaluations. Diffusion-weighted imaging (DWI) with multiple b-values, however, is superior in probing the movement of water molecule in breast tissue [3]. DWI has been increasingly incorporated into breast MRI protocols worldwide [4–6]. Meanwhile, several non-Gaussian diffusion models based on DWI, including the monoexponential model (apparent diffusion coefficient, ADC) [7], biexponential model [8, 9], stretched-exponential model (SEM) [10], statistical model (SM) [11], continuous-time random walk (CTRW) model [12] and diffusional kurtosis imaging (DKI) [13], have been developed to reveal the structures and background of the underlying tissue.

MRI parameters have recently been a hotspot in the analysis and diagnosis of breast lesions [14]. Diagnostic accuracy and efficiency have been improved via a platform of multiple diffusion imaging models to differentiate high- and low-grade brain tumors in adults and children [15–17]. Therefore, a platform with multiple parameters and models could also be applied to the breast. Through multiple DWI parameters with multiple b-values, the accuracy of the diagnosis and evaluation of breast lesions can be further improved, and unnecessary invasive diagnostic and treatment methods can be avoided.

## Methods

### Patients

Institutional review board approval was not applicable in this retrospective study; the need for acquiring informed patient consent was waived, as the data were deidentified and analyzed anonymously.

All patients routinely underwent a breast MRI examination (including DCE-MRI and DWI) at our institution. They all fulfilled the following inclusion criteria: (a) not pregnant, not breastfeeding, no previous treatment and with lesion(s) in the breast; (b) hospitalized and undergoing surgery; and (c) diagnosis was proven by histopathology. Patients who underwent examination after surgical treatment, those in whom the final histopathological diagnosis was established via image-guided needle biopsy, those with no immunohistochemistry results, those with incomplete MRI images or those with inadequate image quality for analysis were excluded.

Finally, 504 women (mean age, 45.7 years; range, 16–87 years) whose largest lesion was confirmed by histopathology were enrolled for further analysis. The median interval between breast MRI and breast surgery was 3 days (range, 1–13 days).

### MRI examination

T2WI, DCE-MRI and DWI were routinely performed within all women in the prone position, using a 3.0T MRI scanner (Achieva; Philips Healthcare) with a 7-channel phased array breast coil. The parameters of T2WI sequence were as follows: TR/TE = 1250/70 msec, 36 slices, field of view (FOV) = 280*340 mm, matrix size = 352*423, 1 NEX, slice thickness = 3.85 mm, acquisition time = 1.5 min. The contrast agent (gadolinium-based agent Gd-DTPA, Magnevist; Bayer Healthcare, Berlin, Germany) was injected intravenously (0.2 mmol/kg body weight at 3 ml/s), using a power injector, followed by a 20-ml saline flush, to obtain DCE images. The parameters of DCE sequence were as follows: temporal resolution = 9.7 s, TR/TE = 3.2/1.55 msec, 29 slices, slice thickness = 3.0 mm, FOV = 360*360 mm, matrix size = 276*276, a flip angle of 10, acquisition time = 6 min 49 s. Seven b-values (0, 500, 1000, 1500, 2000, 2500, and 3000 s/mm^2^) were acquired in DKI sequence in x, y, and z directions, with parameters as follows: TR/TE = 5088/102 msec, FOV = 350*240 mm, matrix size = 140*94, 1 NEX, 32 slices, slice thickness = 4 mm, ACQ (acquisition) voxel size = 2.5*2.5*4 mm^3^, REC (reconstruction) voxel size = 0.99*0.99*4 mm^3^, acquisition time = 8 min and 8 s. The EPI (Echo Planar Imaging) factor was 47, the scan percentage was 97.9% and the WFS (water fat shift) (pix) / BW (bandwidth) (Hz) was 12.647/34.3.

### Selection of regions of interest (ROIs)

According to breast DCE-MRI, the ROI of the largest breast lesion per patient was drawn half-manually, avoiding hemorrhage and cystic or necrotic areas of lesions, using ITK-SNAP (version 3.8.0, Penn Image Computing and Science Laboratory, Philadelphia, PA; http://www.itksnap.org/). Two dedicated breast radiologists (G.C. and L.S.L.) with 4–5 years of experience in the interpretation of breast MR images drew the ROIs independently using ITK-SNAP without knowledge of the clinicopathological data of all patients. Disagreements between the two observers were resolved through a consensus. If any disagreements between the two observers remained, a third dedicated breast radiologist (L.K.A.) with 25 years of experience in breast MRI reached a final decision.

### Multiple DWI parameters

Voxels at all planes of the largest lesion of each patient were included in the calculation. Two types of features (based on DWI models) were extracted from the primary data. The ROI data were filtered by ranking their R-square value from curve fitting, retaining only the top 95% of voxels from each patient. Selected voxels were not totally the same for all models, but the bias was small enough to be ignored. Fitting on a voxel by voxel basis allowed us to exclude signals with severe noise effect. If the ROI signal was averaged directly, voxels with severe noise effect might be included, but if the voxel by voxel fitting was carried out first, we could screen out the voxels with bad signal through the fitting index, which was more accurate. Finally, mean value of each parameter was averaged at ROI level.

The DWI signals were applied to 6 models, and values from the respective parameters were extracted. For image reconstruction, we used the monoexponentional model described in Eq. 1 ($\varvec{S}={\varvec{S}}_{0}\mathbf{e}\mathbf{x}\mathbf{p}(-\varvec{b}\varvec{A}\varvec{D}\varvec{C})$) to calculate the ADC. Then, the biexponential model was applied as described in Eq. 2: $S={S}_{0}(f\text{exp}\left(-bDf\right)+\left(1-f\right)\text{exp}\left(-bDs\right))$. The SEM model was calculated via Eq. 3: $\varvec{S}={\varvec{S}}_{0}\mathbf{e}\mathbf{x}\mathbf{p}(-{\left(\varvec{b}\times \varvec{D}\varvec{D}\varvec{C}\right)}^{\varvec{\alpha }}$. We used Eq. 4 $\left( S={S}_{0}{E}_{{\alpha }_{c}}\left(-{\left(b{D}_{m}\right)}^{{\beta }_{c}}\right)\right),$ represented in the Mittag-Leffler function, to describe the CTRW model. The final two models were the SM, described by Eq. 5 $( \varvec{S}={\varvec{S}}_{0}\mathbf{e}\mathbf{x}\mathbf{p}\left(-\varvec{b}\varvec{A}\varvec{D}{\varvec{C}}_{\varvec{S}}+\frac{1}{2}{\varvec{\sigma }}^{2}{\varvec{b}}^{2}\right))$ and DKI, described by Eq. 6 $( \varvec{S}={\varvec{S}}_{0}\mathbf{e}\mathbf{x}\mathbf{p}\left(-\varvec{b}{\varvec{D}}_{\varvec{K}}+\frac{1}{6}{\varvec{b}}^{2}{\varvec{D}}_{\varvec{K}}^{2}\varvec{K}\right))$.

**Fig. 1:**
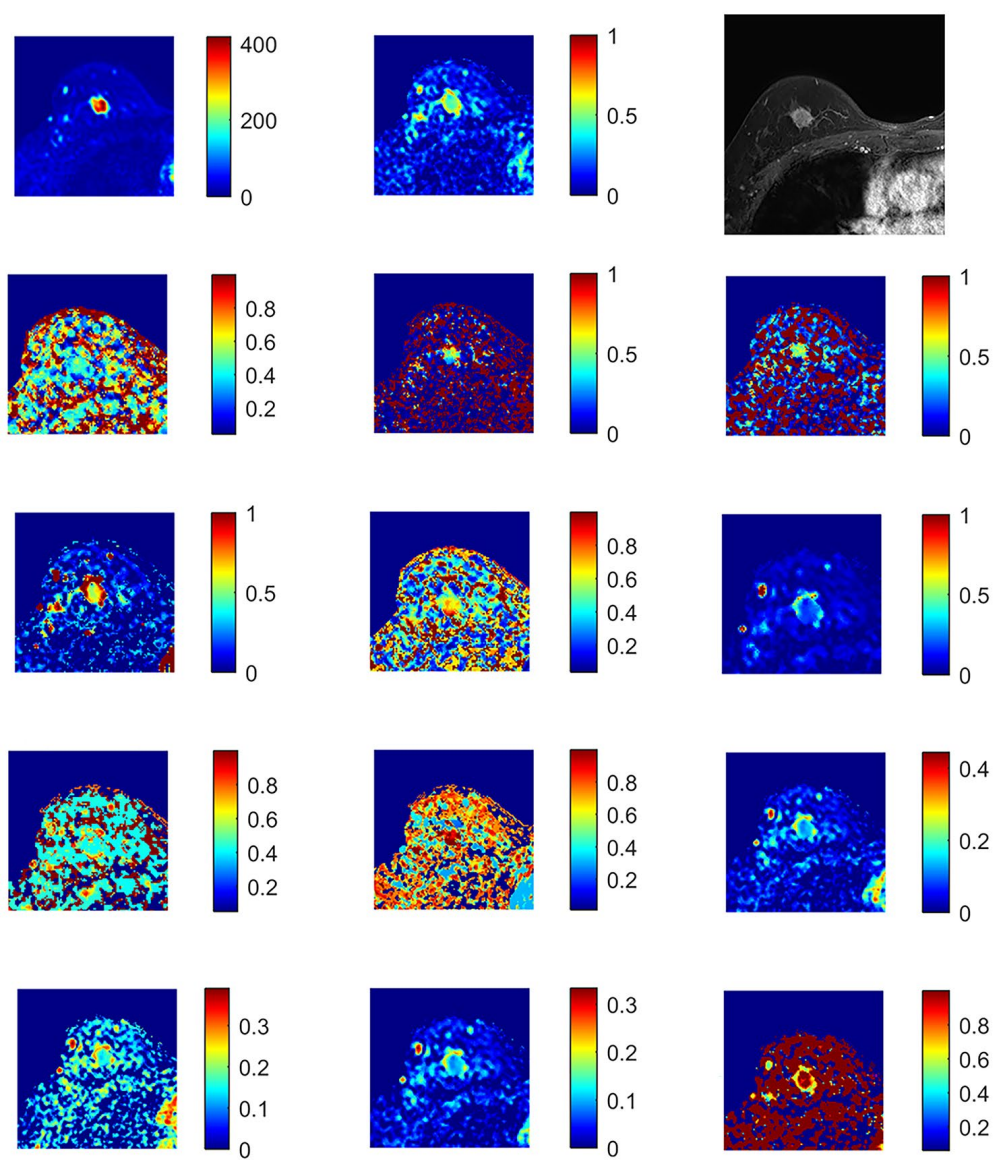
Invasive carcinoma of the right breast: from top to bottom and from left to right, 14 maps of DWI and DCE. b0 map with the ROI (white arrow); ADC map; DCE; f map; Ds map; Df map; DDC map; alpha map; Dc map; alphac map; betac map; ADCS map; sigma map; Dk map and k map

**Fig. 2:**
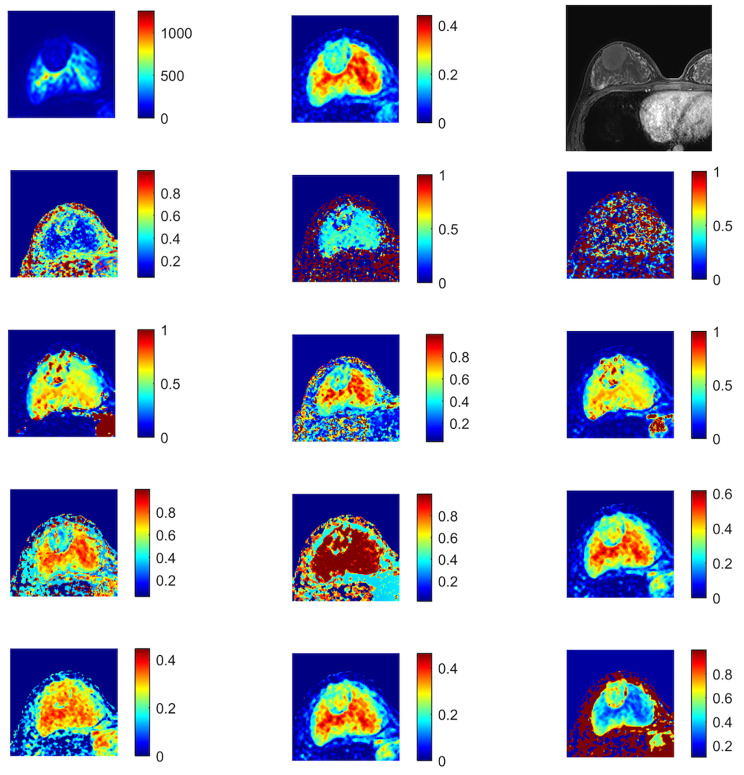
Fibroadenoma of the right breast: from top to bottom and from left to right, 14 maps of DWI and DCE. b0 map with the ROI (white arrow); ADC map; DCE; f map; Ds map; Df map; DDC map; alpha map; Dc map; alphac map; betac map; ADCS map; sigma map; Dk map and k map

The average ADC from the monoexponentional model; perfusion fraction (ƒ), fast diffusion coefficient (D_f_), and slow diffusion coefficient (D_s_) of the biexponential model; distributed diffusion coefficient (DDC), alpha (α) of the SEM; anomalous diffusion coefficient (D_c_) and temporal and spatial heterogeneity parameters (α_c_ and β_c_) of a simplified CTRW model; diffusivity (Dk), kurtosis (K) of DKI; the position of the distribution maxima (ADCs) and width of the diffusion coefficient distribution (sigma) of the SM were calculated (Figs. 1 and 2).

The parameters of the biexponential, SEM and CTRW models were estimated by applying Eq. 2, Eq. 3, and Eq. 4 separately to the acquired b values on a voxel-by-voxel basis with the Levenberg-Marquardt method. The other models were realized by using quadratic polynomial fitting methods. MATLAB (matrix & laboratory) was used to analyze the data and produce maps.

### Histopathology

For all patients, the final histopathological diagnosis was established surgically by paraffin sectioning. There were 265 benign lesions (mean size: 17 mm), mostly fibroadenoma, breast hyperplasia and mastitis lesions, and 239 malignant lesions (mean size: 26 mm), including infiltrating ductal or lobular carcinomas, ductal carcinomas in situ, solid papillary carcinomas, mucinous cancer, and others. Nuclear staining of ≥ 1% of the tumor cells was defined as estrogen receptor (ER) or progesterone receptor (PR) positive. When the expression of HER2 was (3+), it was considered positive; when HER2 expression was (2+), fluorescence in situ hybridization (FISH) detection of gene amplification was required; if the gene was amplified, it was considered positive, and vice versa. Molecular subtypes were determined as follows: Luminal A: ER and PR(+), Ki-67 level less than 20%, and human epidermal growth factor receptor type 2 (HER2) negative; Luminal B (HER2 negative): ER(+) and HER2 negative (PR ˂20% or Ki-67 ≥ 20%); Luminal B (HER2 positive): ER(+), HER2 positive (PR ˂20% or Ki-67 ≥ 20%); HER2 enriched (nonluminal): ER (−), PR (−) and HER2 positive; and triple-negative breast cancer (TNBC): ER, PR and HER2 all negative [18].

### Statistical analysis

The Mann-Whitney U-test and Student’s t test were used for comparisons between the two groups. Receiver operating characteristic (ROC) curves of each parameter, each model (combining two or three parameters) and all 13 parameters combined were generated. Immunohistochemistry and molecular subtype analyses of malignant breast lesions were also conducted. P < 0.05 was considered statistically significant in all tests, and all statistical analyses were performed using SPSS statistical software (v. 22.0, Mariakerke, Belgium).

## Results

A total of 504 lesions, each of which was the largest breast lesion in 504 women, were assessed: 239 were malignant tumors (mean size 26 mm; range 5–90 mm), and 265 were benign lesions (mean size 17 mm; range 2–90 mm).

### Comparisons of all 13 parameters between the benign and malignant groups

**Fig. 3:**
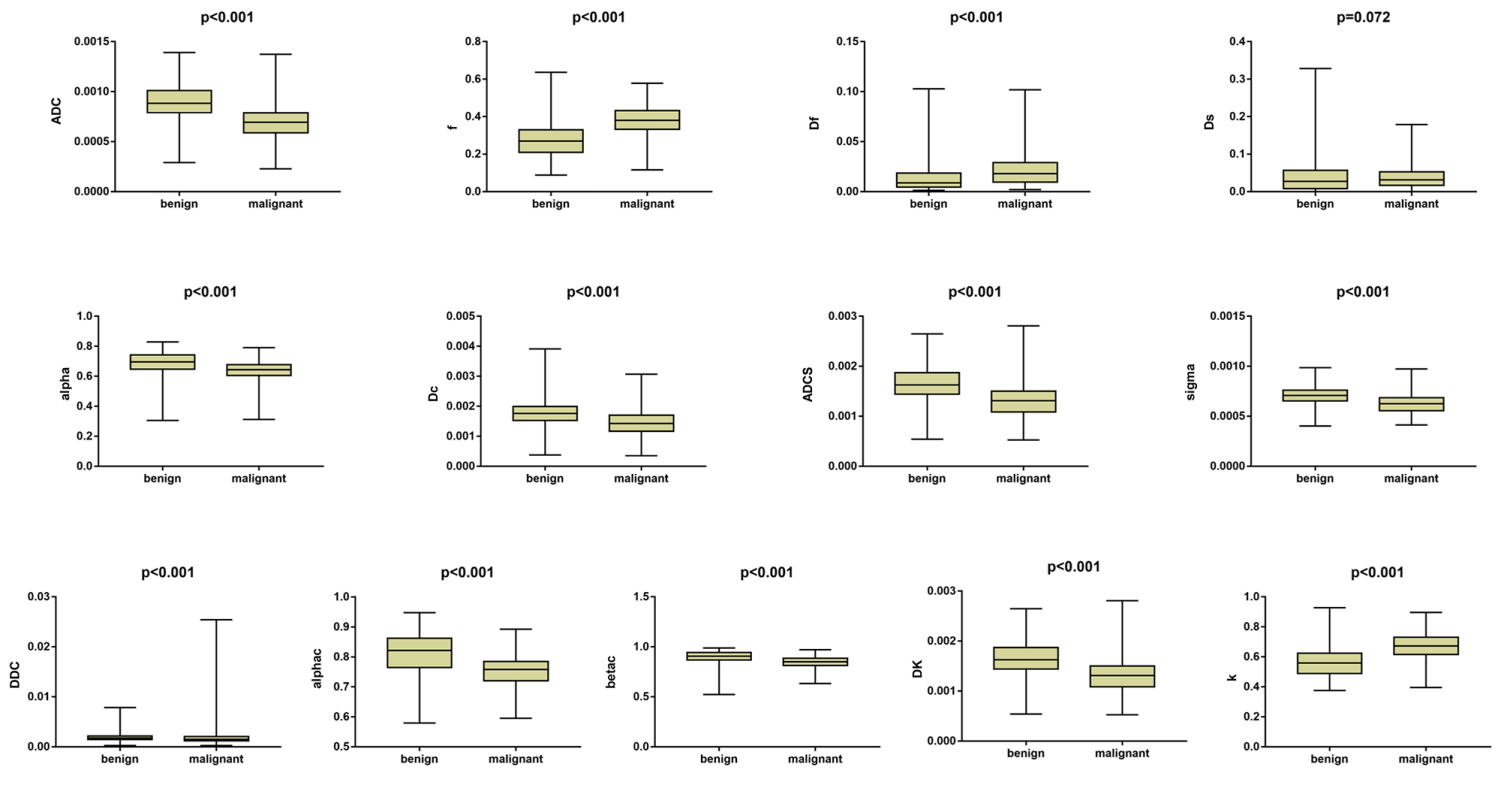
Boxplot distribution of 13 parameters by using multiple b-value DWI in breast lesions

Student’s t test was used to compare 6 parameters (ADC, f, alphac, ADCS, sigma, and DK), and the Mann-Whitney U test was used to compare 7 parameters (Df, Ds, DDC, alpha, Dc, betac, and k) after determining normality. The values of 9 parameters (ADC, DDC, alpha, alphac, Dc, betac, ADCS, sigma, and DK) of the benign group were significantly higher than those of the malignant group. The malignant group exhibited relatively higher f, Df and k values than the benign group. However, the difference of Ds between benign and malignant groups derived from the biexponential model was not statistically significant (0.027 vs. 0.031, p = 0.072). The above results are shown in Table 1; Fig. 3.

**Table 1:** Comparisons of all 13 parameters between the benign and malignant groups

Parameter (mm^2^/sec)	Benign	Malignant	P value
**ADC(×10** ^**− 3**^ **)**	0.894 ± 0.184	0.703 ± 0.161	< 0.001
**f**	0.276 ± 0.092	0.375 ± 0.081	< 0.001
**Df(×10** ^**− 3**^ **)**	8.731(4.472, 18.384)	17.929(9.395, 29.085)	< 0.001
**Ds(×10** ^**− 3**^ **)**	27.338(8.053, 56.603)	31.498(16.506, 52.706)	0.072
**DDC(×10** ^**− 3**^ **)**	1.769(1.473, 2.151)	1.494(1.207, 2.066)	< 0.001
**alpha**	0.697(0.648, 0.742)	0.645(0.606, 0.678)	< 0.001
**alphac**	0.809 ± 0.072	0.753 ± 0.054	< 0.001
**Dc(×10** ^**− 3**^ **)**	1.755(1.523, 1.994)	1.426(1.169, 1.701)	< 0.001
**betac**	0.907(0.868, 0.943)	0.850(0.814, 0.886)	< 0.001
**ADCS(×10** ^**− 3**^ **)**	1.653 ± 0.344	1.325 ± 0.330	< 0.001
**sigma(×10** ^**− 3**^ **)**	0.707 ± 0.088	0.627 ± 0.093	< 0.001
**DK(×10** ^**− 3**^ **)**	1.653 ± 0.344	1.325 ± 0.330	< 0.001
**k**	0.559(0.488, 0.624)	0.672(0.615, 0.731)	< 0.001

Note: Student’s t test was used to compare 6 parameters (ADC, f, alphac, ADCS, sigma, and DK),which was expressed by mean ± standard differences, and the remaining seven parameters are expressed by the median ± quartile using Mann-Whitney U test

### Diagnostic effectiveness of independent or combined parameters

**Fig. 4:**
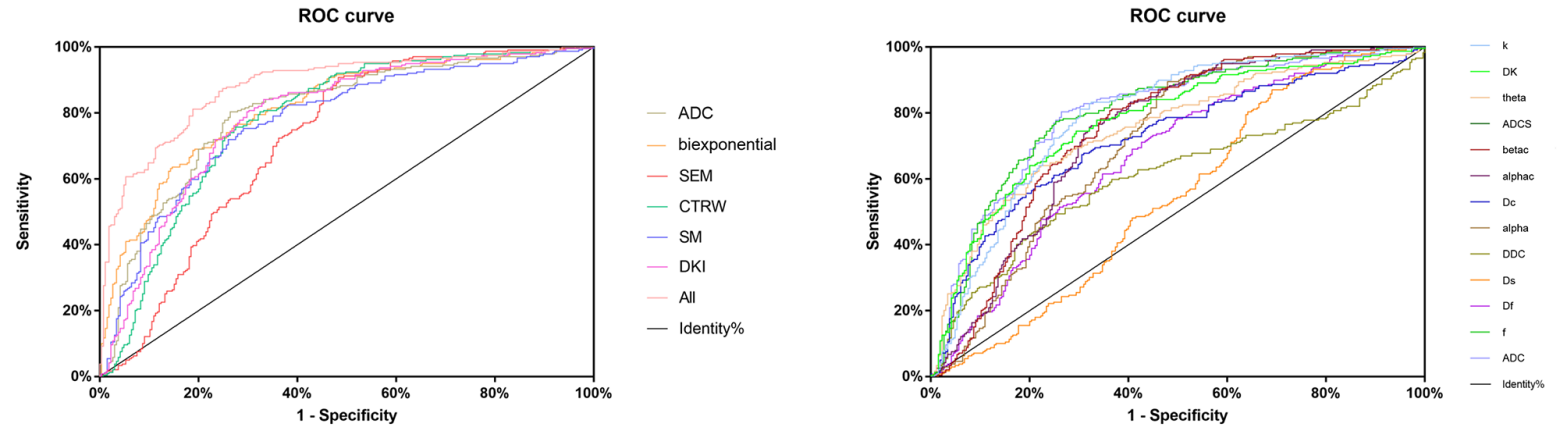
ROC curves were drawn to assess the diagnostic performance of the independent (**b**) and combined (**a**) parameters for discriminating malignant and benign lesions

The results of the ROC curve analysis of each parameter, each model and the combination of all 13 parameters are shown in Table 2; Fig. 4. Among all 13 independent parameters, alpha demonstrated the highest sensitivity (89.5%) with a cutoff value of 0.694, while sigma demonstrated the highest specificity (77.7%) with a cutoff value of 0.651 × 10^− 3^. ADC and f had the highest AUC of 0.803 (95% CI, 0.764–0.842) for discriminating breast cancers from benign lesions.

**Table 2:** ROC curve analysis of independent or combined parameters

	Sensitivity	Specificity	Threshold	AUC	95% CI
**ADC**	0.803	0.736	0.810 × 10^− 3^	0.803	0.764–0.842
**f**	0.774	0.747	0.324	0.803	0.764–0.842
**Df**	0.711	0.574	0.011	0.673	0.626–0.720
**Ds**	0.870	0.309	0.011	0.546	0.496–0.597
**DDC**	0.494	0.747	1.484 × 10^− 3^	0.609	0.558–0.659
**alpha**	0.895	0.521	0.694	0.714	0.668–0.759
**Dc**	0.678	0.691	1.587 × 10^− 3^	0.721	0.676–0.766
**alphac**	0.762	0.675	0.786	0.742	0.699–0.786
**betac**	0.812	0.634	0.893	0.753	0.710–0.796
**ADCS**	0.745	0.706	1.492 × 10^− 3^	0.775	0.734–0.816
**sigma**	0.640	0.777	0.651 × 10^− 3^	0.753	0.711–0.796
**DK**	0.745	0.706	1.492 × 10^− 3^	0.775	0.734–0.816
**k**	0.812	0.698	0.604	0.790	0.750–0.830
**biexponential**	0.690	0.808	0.535	0.817	0.780–0.854
**SEM**	0.908	0.517	0.406	0.719	0.674–0.764
**CTRW**	0.757	0.725	0.460	0.781	0.740–0.822
**DKI**	0.841	0.668	0.411	0.792	0.752–0.831
**SM**	0.753	0.709	0.467	0.776	0.735–0.817
**All**	0.874	0.751	0.393	0.882	0.852–0.912

Among the 6 independent models, the SEM in which DDC and alpha were combined demonstrated the highest sensitivity (90.8%) with a cutoff value of 0.406, while f, Df and Ds derived from the biexponential model demonstrated the highest specificity (80.8%) and the highest AUC of 0.817 (95% CI, 0.780–0.854) with a cutoff value of 0.535.

The highest AUC was achieved when all 13 parameters were combined (0.882, 95% CI, 0.852–0.912), which was higher than that of each independent parameter or model.

### Immunohistochemistry and molecular subtype analyses of malignant tumors

Among the 239 malignant tumors (mean size 26 mm; range 5–90 mm), the 13 parameters were compared with ER, PR, HER2, and Ki-67 expression and tumor size via point-biserial correlation and Spearman’s correlation analyses. Except for DDC, all 12 parameters were weakly positively or negatively correlated with ER. Except for DDC and sigma, all 11 parameters were weakly positively or negatively correlated with PR. Three parameters (Ds, alpha and alphac) were weakly positively or negatively correlated with HER2. High Ki-67 expression and tumor size were weakly positively or negatively correlated with Df, Ds and alpha. In addition, betac was weakly positively correlated with tumor size (r = 0.141, p = 0.036) (Table 3).

**Table 3:** Correlation analyses between 13 parameters and prognostic factors

Parameter		ER	PR	HER2	Ki-67	Size
**ADC**	r	-0.204	-0.160	0.072	-0.012	0.095
	p	0.002	0.017	0.288	0.864	0.159
**f**	r	0.164	0.133	-0.019	0.028	-0.031
	p	0.014	0.048	0.774	0.679	0.641
**Df**	r	0.190	0.206	-0.125	-0.164	-0.204
	p	0.004	0.002	0.063	0.015	0.002
**Ds**	r	0.159	0.143	-0.136	-0.273	-0.210
	p	0.017	0.033	0.043	< 0.001	0.002
**DDC**	r	-0.025	0.043	0.091	-0.050	0.006
	p	0.706	0.528	0.179	0.456	0.928
**alpha**	r	-0.222	-0.212	0.167	0.202	0.182
	p	0.001	0.001	0.013	0.002	0.007
**Dc**	r	-0.201	-0.138	0.114	-0.008	-0.011
	p	0.003	0.040	0.090	0.911	0.871
**alphac**	r	-0.236	-0.207	0.177	0.064	0.038
	p	0.000	0.002	0.008	0.345	0.572
**betac**	r	-0.211	-0.209	0.100	0.126	0.141
	p	0.002	0.002	0.136	0.062	0.036
**ADCS**	r	-0.197	-0.145	0.054	-0.024	0.052
	p	0.003	0.030	0.420	0.719	0.442
**sigma**	r	-0.148	-0.102	0.005	-0.066	< 0.001
	p	0.027	0.129	0.945	0.328	0.999
**Dk**	r	-0.197	-0.145	0.054	-0.024	0.052
	p	0.003	0.030	0.420	0.719	0.442
**k**	r	0.183	0.133	-0.074	0.012	-0.081
	p	0.006	0.048	0.273	0.863	0.229

The Kruskal-Wallis H test and analysis of variance (ANOVA) were used to determine whether 13 parameters exhibited significant differences among the 5 molecular subtypes of breast cancer. Eight parameters (Df, Dc, alphac, betac, ADC, alpha, ADCS and DK) were significantly different among the 5 molecular subtypes of breast cancer (P < 0.05) (Table 4). After Bonferroni correction, 6 parameters were significantly different among pairwise comparisons (Table 5). ADC, Dc, ADCS and DK were significantly larger in the TNBC group than in the Luminal B (HER2-negative) group. The HER2-enriched group had higher ADC, alpha, alphac, ADCS and DK values than the Luminal B (HER2-negative) group. alpha and alphac were significantly smaller in the Luminal A group than in the HER2-enriched group.

**Table 4:** Statistical differences among the 5 molecular subtypes of breast cancer

Parameter	F	P value
**k**	2.076	0.085
	**Chi-square**	
**f**	8.901	0.064
**Df**	9.871	0.043
**Dc**	14.044	0.007
**alphac**	18.883	0.001
**betac**	12.853	0.012
**sigma**	9.313	0.054
**ADC**	15.925	0.003
**Ds**	9.444	0.051
**DDC**	3.391	0.495
**alpha**	19.827	0.001
**ADCS**	15.642	0.004
**DK**	15.642	0.004

**Table 5:** Statistical differences among pairwise comparisons

Parameter (mm^2^/sec)	Molecular subtype	Median	P value
**ADC**	Luminal B (HER2 negative) vs. TNBC	0.646 × 10^− 3^ vs. 0.758 × 10^− 3^	0.036
	Luminal B (HER2 negative) vs. HER2 enriched	0.646 × 10^− 3^ vs. 0.747 × 10^− 3^	0.021
**alpha**	Luminal A vs. TNBC	0.630 vs. 0.665	0.027
	Luminal A vs. HER2 enriched	0.630 vs. 0.672	0.006
	Luminal B (HER2 negative) vs. HER2 enriched	0.637 vs. 0.672	0.015
**Dc**	Luminal B (HER2 negative) vs. TNBC	1.370 × 10^− 3^ vs. 1.581 × 10^− 3^	0.036
**alphac**	Luminal A vs. HER2 enriched	0.730 vs. 0.778	0.004
	Luminal B (HER2 negative) vs. HER2 enriched	0.749 vs. 0.778	0.010
**ADCS**	Luminal B (HER2 negative) vs. TNBC	1.209 × 10^− 3^ vs. 1.456 × 10^− 3^	0.029
	Luminal B (HER2 negative) vs. HER2 enriched	1.209 × 10^− 3^ vs. 1.423 × 10^− 3^	0.027
**DK**	Luminal B (HER2 negative) vs. TNBC	1.209 × 10^− 3^ vs. 1.456 × 10^− 3^	0.029
	Luminal B (HER2 negative) vs. HER2 enriched	1.209 × 10^− 3^ vs. 1.423 × 10^− 3^	0.027

## Discussion

As personalized therapies continue to develop in the field of breast tumor treatment, accurate tumor characterization is essential for further appropriate treatment.

The application of 13 parameters into the characterization of breast lesions Malignant breast lesions usually demonstrate dense cellularity and a complex tissue microenvironment, resulting in more deviation from a Gaussian distribution with higher diffusional kurtosis than benign breast lesions [19]. Therefore, the ADC [20] and DK values in malignant lesions were significantly lower than those in benign lesions, while k was the opposite, consistent with our study and many other studies [4, 21, 22], further proving their solid position in tumor characterization and improving their possibility of clinical application.

The biexponential model was proposed to evaluate both tissue diffusivity and tissue microcapillary perfusion. f and Df exhibited relatively higher values in the malignant group than in the benign group, but Ds in the benign and malignant groups was not significantly different, inconsistent with the results from other studies [23, 24]. It may be related to the choice of b values since high b values increase the accuracy in Ds estimation and low b values improve the measurements of perfusion-related parameters [25].

DDC reflects the mean intravoxel diffusion rate, and alpha is an intravoxel diffusion heterogeneity index, providing important information in differentiating malignant from benign lesions, which was proven in our statistical results.

The CTRW model and SM have been applied in very few clinical studies thus far. M.M. [26] found that the CTRW diffusion model offers potential for exploring tumor structural heterogeneity at a subvoxel level and assessing glioma malignancy. The SM was used to monitor the treatment response (e.g., to explore the possibility of assessing early response to chemotherapy in patients with colorectal liver metastasis) [27]. In our study, the CTRW and SM model, respectively, in the benign group were significantly higher than those in the malignant group. This finding might be explained by malignant breast lesions manifesting extensive tissue heterogeneity and structural complexity, theoretically resulting in elevated non-Gaussian diffusion characteristics [12]. Therefore, this article innovatively applied these two models to breast masses and further confirmed their value in tumor characterization, laying a solid foundation for its clinical application.

### Diagnostic performance of all parameters

ADC exhibited a higher AUC (0.803) than Dk, k and DKI model, inconsistent with the results reported by Huang, Y [21]. According to our findings, the DKI model did not perform superior to ADC in the clinic [19], probably because our study included 50 patients with non-mass enhancement (NME). Many studies have specifically excluded NME since they were harder to segment, however, this type of lesion was very common in clinical practice, which made this article more suitable for clinical promotion. f and ADC had a high AUC of 0.803, but the biexponential model achieved a higher AUC (0.817) than the ADC and demonstrated the highest specificity (80.8%). These findings indicate that the biexponential model performs better than the ADC with regard to diagnostic accuracy [28], which laid the foundation for the further routine application of biexponential in clinical practice.

The ROC curve analysis suggested that the independent parameter alpha demonstrated the highest sensitivity and relatively low specificity. When alpha was combined with DDC (SEM), the sensitivity was further improved (90.8%). Although the SEM did not outperform ADC in diagnostic accuracy, inconsistent with the results reported by Chen, B.Y., et al. [29], it still showed promising prospects in breast cancer diagnosis, especially the extremely high sensitivity in breast cancer detection. In another word, the high sensitivity of the SEM model (90.8%) was expected to be applied into the clinical screening of breast cancer. Similarly, the high specificity of the biexponential model (80.8%) was expected to reduce the misdiagnosis rate of breast cancer in clinical work, to minimize the overtreatment of patients with benign breast lesions. Karaman, M.M. also reported that the CTRW model provided comparable performance with the ADC; [17] Although the CTRW model and the SM model did not show the same superiority in breast lesions in this study, they still showed high diagnostic performance and promising prospects.

Many articles have made comparisons between different models, it is inevitable that the diagnostic performance of one certain model or parameter is better than the other one, due to the influence of many factors such as data collection and regional differences, which makes it difficult to unify the diagnostic standards. In our study, this problem may be solved when all 13 parameters were combined, as the highest AUC (0.882) was achieved, when the diagnostic performance in distinguishing benign and malignant breast lesions was the best. This result suggests that compared to the traditional and regularly used ADC, the combination of more newly applied parameters may provide additional valuable information on changes in the microenvironment and tumor characterization. If all 13 parameters were taken into account, the characterization accuracy of breast masses would be very high, which could be a great improvement for actual clinical applications and further artificial intelligence.

### Correlation between parameters and prognostic factors

ER- or PR-positive tumors tend to be weakly negatively correlated with ADC values. Charles S Springer Jr [30] deduced this correlation as a result of different levels of cell membrane permeability. Alexey Surov [31] suggested that the ADC had a weak correlation with high Ki-67 expression, with poor diagnostic accuracy (sensitivity 64%, specificity 50%). However, ADC, K, and Dk had no correlation with Ki-67 or HER2 expression in our study. Therefore, ADC has certain significance in predicting ER and PR status in breast cancer, but not in predicting proliferative activity.

Suo et al. [23] found a correlation only between Df and high Ki-67 expression, and, Ds, f with ER expression. But we found more: Each parameter of the biexponential model was significantly correlated with the expression of hormonal receptors (ER and PR) and all correlations were positive; Ds were weakly negatively correlated with high Ki-67 expression [32] and HER2 expression. Although there was the possible utility of the biexponential model in prognostic factors prediction of breast lesions, the possibility seemed relatively small.

Suo, S [23] reported that DDC was significantly correlated with ER expression. There are relatively few clinical studies on this model, so it is difficult to reach a unified conclusion. Our findings, on the other hand, suggested more: alpha was correlated with ER, PR, HER2 and Ki-67, which meant that alpha had the potential to provide useful information on the genetic properties and proliferative activity of breast tumors. The correlation analyses between CTRW, SM model and prognostic factors suggested that these two models had potential to provide certain guiding suggestions on individualized treatment of breast tumors.

Based on correlation analysis, using all these parameters and models to predict prognostic factors in breast cancer has not achieved positive results.

### Differences of parameters among different molecular subtypes

The stratification of breast cancers by subtype is important for treatment planning. None of the 13 parameters were significantly different between the Luminal B (HER2-positive) group and the other 4 groups. Among the 6 parameters with significant differences among the 5 molecular subtypes of breast cancer, the Luminal A group and Luminal B (HER2 negative) group had relatively low values, and the HER2-enriched group and TNBC group had relatively high values.

Whether the ADC values in the Luminal A group or Luminal B (HER2 negative) group were lower than those in other subtypes was unclear in other researches [33, 34]. According to our findings, the ADC values in the Luminal B (HER2 negative) group were lower. The ADC values in the TNBC or HER2-enriched group were higher than those in the Luminal B (HER2 negative) group, consistent with results from some other studies [35, 36].

Zhao, M. found that IVIM parameters exhibited significant differences between different tumor subtypes [37]. Unfortunately, our results do not support such significant differences. This inconsistency could be caused by larger sample sizes of breast malignancy and different choices of b values in our study. Our study found that Dc and alphac from the CTRW model, ADCS from the SM, alpha from the SEM and Dk from DKI were significantly different between different molecular subtypes of breast cancer. There has not been such a discovery in any other research on breast cancer, which might lay the foundation for the feasibility of using multiple parameters to further predict molecular subtypes. Although it is just a small step, with further large-scale researches, the clinical value can be further explored.

Although it takes a small amount of additional scanning time to obtain multiple b-value MR images, multiple parameters and models derived from post-processing can be easily attained within a short time. C.X.L. [38] explored the diagnostic value of multiple b-value DWI in the characterization of breast lesions with 79 patients in total, which was far less than our large cohort study. Furthermore, the ROI was placed on the largest tumor transverse-sectional level in their research, while voxels at all planes of the largest lesion of each patient were included in our calculation in order to reduce deviation. Moreover, the research of C.X.L. mainly focused on three models of ADC, biexponential diffusion model and stretched-exponential diffusion model, while in our study, there were thirteen parameters of six models. In addition, the application of advanced diffusion models in prognostic factors and molecular subtypes of breast malignancy was also explored in our study. Finally, the highest AUC of all parameters in their research was not as high as the combination of all parameters in this paper (80.7% vs. 88.2%). Therefore, these points further reflected that our research was quite meaningful and expected to further promote the application of multiple parameters and models into clinical practice.

More importantly, these derived indicators are essential as biomarkers for the diagnosis and evaluation of breast masses in the discussion part of this paper, especially the three emerging models of SM, SEM, and CTRW manifested unique value when applied to the breast. Therefore, the efficiency and quality of diagnosis can be further improved, in line with the necessary condition for its promotion and application into clinical practice. There is hope that all masses on breast MRI can be routinely analyzed with multiple parameters and models in the future, so as to make rapid, accurate and standardized characterization of breast masses and further predict prognostic factors and molecular subtypes of malignant breast tumors.

There are several potential limitations to this study. First, this was a single-center study, and the recruitment of patients may have been affected by geographical factors such as living environment and lifestyle. Second, data acquisition and processing should be standardized if these parameters were expected to be applied to clinical diagnosis because scanning methods, and the selection of b values and MRI systems vary in each center.

## Summary and conclusions

All 13 parameters, independent or combined, provide tremendously valuable information in distinguishing malignant from benign breast lesions. These new parameters are promising auxiliary diagnostic tools for improving the diagnostic accuracy of breast lesions, and these new parameters have limited meaning for predicting prognostic factors and molecular subtypes of malignant breast tumors.

## Data Availability

The datasets generated during and analyzed during the current study are not publicly available due to protection of study participant privacy, but are available from the corresponding author on reasonable request.

## Abbreviations

DWI: Diffusion-weighted imaging
MRI: Magnetic resonance imaging
DCE: Dynamic contrast-enhanced
WHO: World Health Organization
ER: Estrogen receptor
PR: Progesterone receptor
HER2: Human epidermal growth factor receptor type 2
ADC: Apparent diffusion coefficient
SEM: Stretched-exponential model
SM: Statistical model
CTRW: Continuous-time random walk
DKI: Diffusional kurtosis imaging
ROI: Regions of interest
ROC: Receiver operating characteristic
ACQ: Acquisition
REC: Reconstruction
EPI: Echo Planar Imaging
WFS: Water fat shift
BW: Bandwidth
